# Tracheobronchial stenosis as a rare extraintestinal manifestation of ulcerativecolitis: a case series with different therapeutic approaches

**DOI:** 10.3389/fmed.2026.1816032

**Published:** 2026-06-03

**Authors:** Umberto Zanini, Giovanni Franco, Sofia Maria Mazzotta, Luca Geroli, Francesca Bono, Giuseppe Paciocco, Paola Faverio, Fabrizio Luppi, Almerico Marruchella

**Affiliations:** 1 Respiratory Unit, Fondazione IRCCS San Gerardo dei Tintori, Monza, Italy; 2 Department of Medicine and Surgery, University of Milano-Bicocca, Milan, Italy; 3 Department of Pathology, Fondazione IRCCS San Gerardo dei Tintori, Monza, Italy

**Keywords:** bronchial stenosis, bronchoscopy, case series, IBD, ulcerative colitis

## Abstract

**Background:**

Ulcerative colitis (UC) is a chronic inflammatory bowel disease characterized by extraintestinal manifestations in nearly half of patients. Although pulmonary involvement has traditionally been considered rare, with a reported prevalence of less than 1%, recent studies indicate that subclinical airway abnormalities may occur in 40–60% of cases. Tracheobronchial stenosis is an uncommon but clinically significant extraintestinal manifestation that causes considerable diagnostic and therapeutic challenges.

**Case series:**

This report presents three female patients with UC who developed significant bronchial stenosis, predominantly affecting the left main bronchus. Notably, a temporal dissociation between intestinal and respiratory disease activity was observed; in two cases, airway symptoms developed years after the initial UC diagnosis, and in one case, symptoms appeared following total colectomy during intestinal disease remission. Histopathological examination consistently revealed chronic lymphoplasmacytic infiltrates and mucosal edema.

**Management:**

Therapeutic responses varied considerably, ranging from active inflammation to irreversible fibrotic remodeling. One patient, unresponsive to systemic corticosteroids, required rigid bronchoscopy with balloon dilatation to restore airway patency. Another patient experienced rapid improvement with high-dose corticosteroids administered during a UC flare, while the third demonstrated partial improvement with persistent cicatricial stenosis.

**Conclusion:**

Early identification of tracheobronchial involvement in UC is important to prevent irreversible pulmonary damage. Given the rarity of this manifestation and the absence of standardized treatment protocols, multidisciplinary management incorporating medical therapy and interventional bronchoscopy is necessary to individualize patient care.

## Introduction

Inflammatory bowel diseases (IBDs) are chronic inflammatory conditions of the gastrointestinal tract. They are characterized by a relapsing–remitting course. The two main forms are ulcerative colitis (UC) and Crohn’s disease (CD). Over the past decade, the incidence of IBD has increased worldwide (1). Currently, IBD affects approximately 7 million individuals globally, with notable geographic differences (2). This disease imposes a substantial burden on patients’ quality of life and on healthcare systems. Disease onset most commonly occurs between 15 and 30 years of age. A second incidence peak is observed between ages 40 and 50 (3). Although the precise pathogenesis of IBD remains unclear, several contributing factors have been identified. These include genetic susceptibility, alterations in the gut microbiota, and environmental influences, all of which may promote chronic intestinal inflammation (4). The pathogenesis of UC-associated airway disease is also thought to involve the “gut–lung axis,” characterized by aberrant immune cell trafficking, systemic inflammation, and microbial translocation (5). Gut-primed lymphocytes may mis-home to the respiratory tract, promoting chronic airway inflammation through the recruitment of neutrophils, macrophages, eosinophils, and other inflammatory cells. Cytokines such as TNF-*α*, IL-17, IL-13, and IL-5 may contribute to epithelial injury, airway remodeling, and progressive fibrotic stenosis (6). In addition, the shared embryologic origin of the gastrointestinal and respiratory epithelium may partially explain the occurrence of parallel inflammatory responses in both systems (7). Notably, nearly half of patients with IBD experience manifestations beyond the gastrointestinal tract. These involve organs such as the joints, skin, eyes, and liver (8).

Pulmonary involvement is an uncommon extraintestinal manifestation. It is frequently underrecognized, which leads to delayed diagnosis and potentially poorer outcomes. The prevalence of thoracic involvement in IBD is estimated to be below 1%, although this is likely an underestimate (9). Recent evidence suggests that 40–60% of patients with IBD may have abnormalities on pulmonary function tests (PFTs) or chest computed tomography (CT) imaging (10). The respiratory conditions most often associated with IBD include chronic bronchitis, subglottic stenosis, bronchiectasis, and bronchiolitis. These conditions may further complicate the identification of IBD-related lung disease. Thoracic manifestations appear to be more frequent in patients with UC compared with other extraintestinal complications of IBD (9). In most cases, respiratory symptoms arise several years after the onset of gastrointestinal disease. In fewer than 15% of patients, pulmonary involvement may coincide with or even precede intestinal symptoms (11). Thoracic manifestations have also been described in patients with UC, particularly following curative colorectal surgery (12).

Early recognition of respiratory involvement in patients with IBD is essential, as it helps prevent the progression of lung disease and improves prognosis (13). Consequently, early detection also guides the selection of appropriate and timely therapeutic strategies (14). Management of IBD-related airway disease may involve only medical treatment or, depending on the severity and extent of airway involvement, may require more invasive procedures such as rigid bronchoscopy (15). To illustrate the varied appearance and treatment of this disease, we present three cases in this series. Each case demonstrates a distinct clinical presentation and therapeutic approach to bronchial involvement in UC, underscoring the need for individualized, multidisciplinary management (Table 1).

**Table 1:** Clinical characteristics, diagnostic findings, and management of three patients with UC-related bronchial stenosis.

Feature	Case 1	Case 2	Case 3
Patient	56-year-old female	28-year-old female	33-year-old female
UC history & status	Diagnosed 1988; total colectomy (2002); clinical remission at respiratory onset	Diagnosed 2023; symptoms followed a UC flare (bloody diarrhea) by 2 weeks	Diagnosed 2009; long-term clinical remission on azathioprine
Respiratory symptoms	Progressive cough, dyspnea, and recurrent infections	Acute dyspnea, cough, and chest tightness	Chronic productive cough and reduced exercise tolerance
Chest CT findings	Narrowing of the left main bronchus (LMB); mucus plugging	Concentric thickening of subglottic trachea and bronchi; tracheal diameter reduced to 7 mm	Bilateral bronchiectasis; diffuse tracheal/bronchial thickening; LMB narrowing
Bronchoscopic appearance	Diffuse edema; critical stenosis of the LMB with purulent secretions	Inflammatory changes; endoluminal protrusions; 50% reduction in LMB caliber	Edema; “cobblestone” appearance of mucosa; passable LMB stenosis
Histopathology	Chronic lymphoplasmacytic infiltrates; epithelial ulceration	Chronic active inflammation with focal erosive changes	Fibrosis, squamous metaplasia, and lymphoplasmacytic infiltrates with eosinophils
Therapeutic approach	Refractory to systemic steroids; Rigid bronchoscopy with balloon dilatation; local gentamicin	High-dose systemic corticosteroids (methylprednisolone 1 mg/kg/day)	Systemic corticosteroids (prednisone 50 mg/day)
Clinical outcome	Immediate restoration of airway patency; stable at 1-year follow-up	Rapid clinical improvement and radiological regression of stenosis	Significant improvement; persistence of residual cicatricial stenosis

## First case report

A 56-year-old Caucasian woman with ulcerative colitis diagnosed in 1988 was referred to our Pulmonology Unit. She presented with progressive cough, dyspnea, and recurrent lower respiratory tract infections. Her medical history included UC-related bronchiectasis since the late 1980s, requiring left lower lobectomy in 1991. She underwent total colectomy in 2002, after which UC remained in clinical remission.

**Figure 1:**
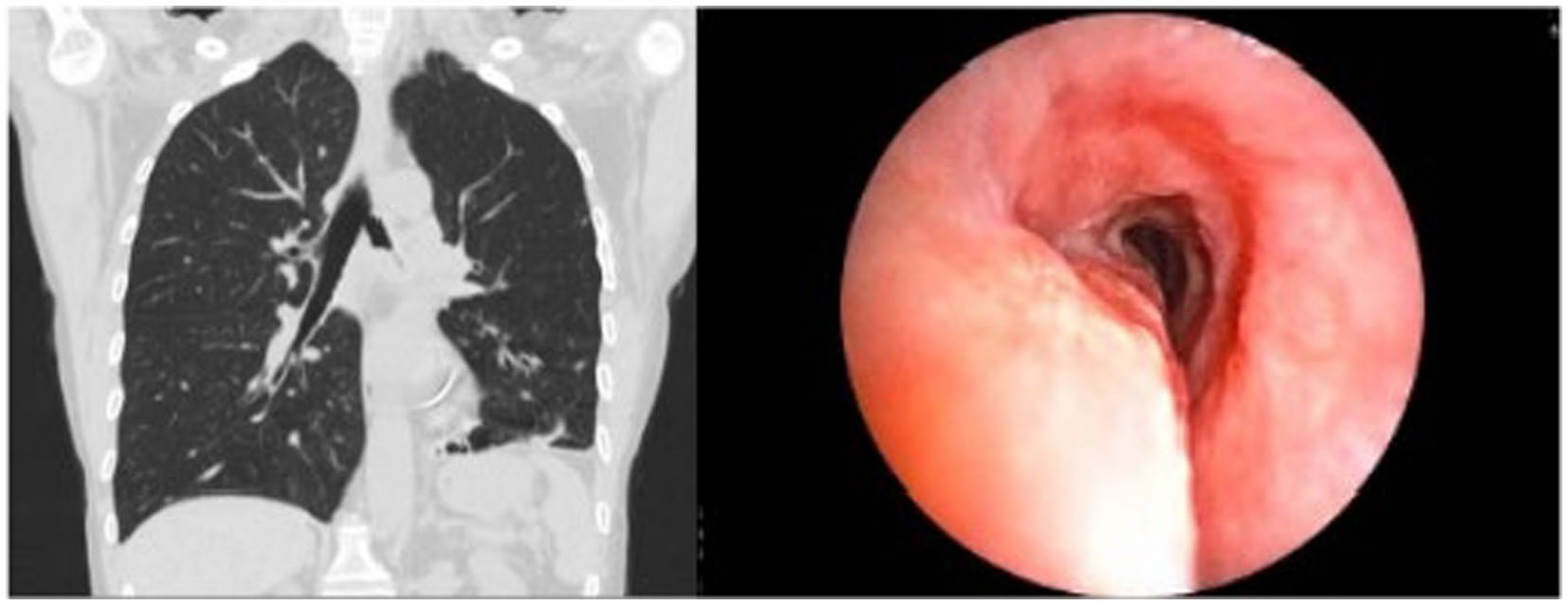
Case 1: Coronal CT scan (left) showing severe stenosis of the left main bronchus, and corresponding bronchoscopic view (right) demonstrating the endoluminal narrowing at the same anatomical site.

Chest computed tomography showed narrowing of the left main bronchus and mucus plugging of peripheral bronchiectasis (Figure 1). These findings were not present on previous imaging. Flexible bronchoscopy revealed diffuse inflammatory and edematous mucosa with critical stenosis of the left main bronchus and purulent secretions (Figure 1). Initial bronchial aspirate cultures were positive for *Pseudomonas aeruginosa*. Targeted antibiotic therapy and high-dose systemic corticosteroids (prednisone 1 mg/kg/day, slow taper) did not result in significant clinical or endoscopic improvement.

**Figure 2:**
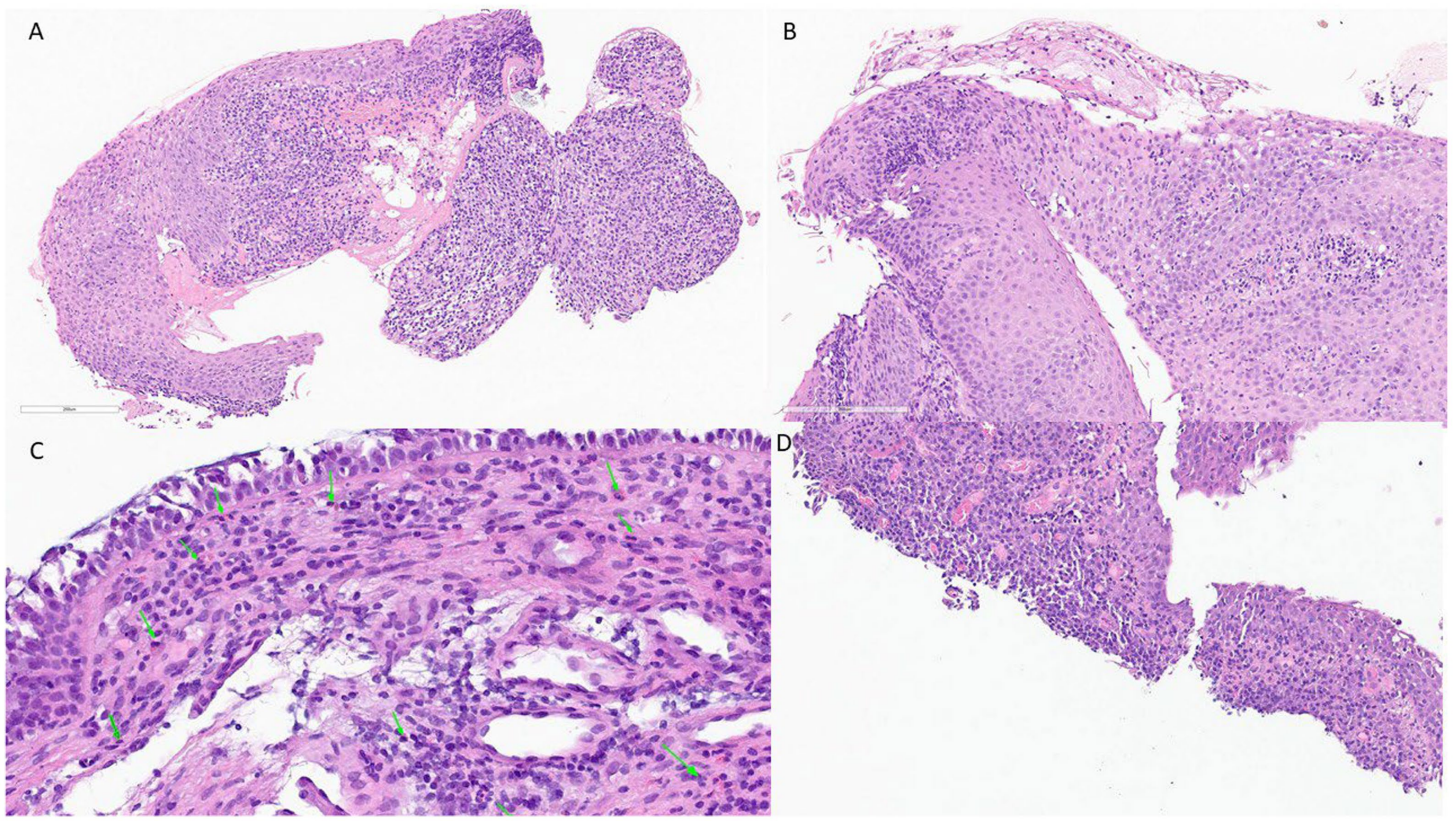
The images (H&E, 10–20×) show, in all three cases for which bronchoscopic biopsy was available, the presence of chronic inflammatory infiltrates consisting of lymphocytes, plasma cells, and a moderate presence of eosinophilic granulocytes [see arrows in panel **(C)**], with moderate-to-severe erosive inflammation and granulation tissue formation. No necrosis or granulomas were identified. Histochemical stains for infectious agents (PAS, Grocott, and Ziehl–Neelsen) were negative in all cases. Panels **(A,B)** correspond to Case 1 (10×), panel **(C)** to Case 2 (20×), and panel **(D)** to Case 3 (10×). A scale bar is shown in panel **(A)**.

Endobronchial biopsies showed dense chronic inflammatory infiltrates predominantly composed of lymphocytes and plasma cells, associated with mucosal edema and focal epithelial ulceration (Figures 2A,B). Areas of active inflammation with erosive changes were also observed. No granulomas, vasculitis, necrosis, or malignant cells were identified. There was no evidence of infection, granulomatous disease, vasculitis, or malignancy. Other causes of bronchial stenosis were excluded. Multidisciplinary discussion led to the diagnosis of bronchial involvement as an extraintestinal manifestation of UC.

Persistent symptoms and critical airway obstruction led to rigid bronchoscopy with balloon dilatation of the left main bronchus. This procedure immediately improved airway patency and respiratory symptoms. During follow-up, recurrent infectious exacerbations required repeated bronchoscopic procedures with local gentamicin administration. At 1 year, the bronchial lumen remained patent, symptoms remained stable, and UC remained in remission with low-dose maintenance corticosteroids.

## Second case report

A 28-year-old woman, a never-smoker, with ulcerative colitis diagnosed in 2023 (symptom onset in 2021), was admitted for the acute onset of progressive dyspnea, cough, and chest tightness. Two weeks before admission, she experienced a flare of UC characterized by bloody diarrhea, treated with intensified oral and topical mesalazine. She had no previous respiratory disease.

Chest CT revealed marked concentric thickening of the subglottic trachea and main bronchi, with significant reduction of the tracheal transverse diameter (minimum 7 mm) and irregular bronchial mucosal profiles. No pulmonary parenchymal involvement or pulmonary embolism was detected. Flexible bronchoscopy showed inflammatory changes of the tracheal mucosa with small endoluminal protrusions and approximately 50% reduction in the caliber of the left main bronchus. Endobronchial biopsies revealed chronic active inflammatory infiltrates composed predominantly of lymphocytes and plasma cells, with focal erosive epithelial changes and scattered eosinophilic granulocytes (Figure 2C).

Microbiological investigations, including bacterial, viral, fungal, and mycobacterial testing, were negative. Given the temporal relationship with UC exacerbation and the exclusion of other etiologies, a diagnosis of acute tracheobronchial involvement related to UC was made.

**Figure 3:**
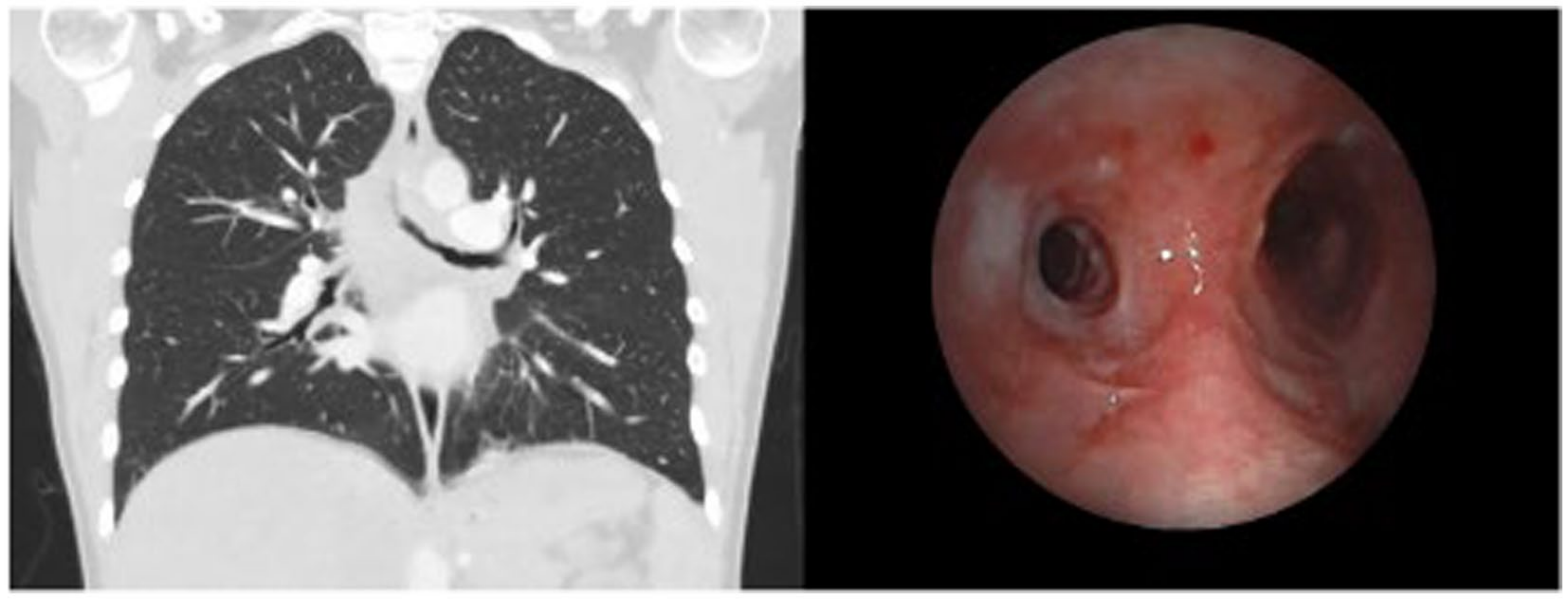
Case 2: Coronal CT scan (left) showing severe stenosis of the left main bronchus, and corresponding bronchoscopic view (right) demonstrating the endoluminal narrowing at the same anatomical site.

High-dose systemic corticosteroid therapy (methylprednisolone, approximately 1 mg/kg/day) was initiated, resulting in rapid clinical improvement and complete weaning from oxygen therapy. The patient was discharged on oral prednisone with a slow tapering schedule. Escalation to biologic therapy for UC management was subsequently considered as a steroid-sparing strategy. A mild cough recurrence occurred during steroid dose reduction, requiring a temporary dose adjustment. At follow-up, she remained clinically stable, with normal oxygen saturation, radiological regression of the stenosis and no recurrence of severe respiratory symptoms (Figure 3). Escalation to biological therapy for UC was planned.

## Third case report

A 33-year-old woman, a never-smoker, with ulcerative colitis diagnosed in 2009, was referred for chronic productive cough, reduced exercise tolerance, and impaired quality of life. UC had been treated with azathioprine until 2024 and was in long-term clinical remission, with no recent intestinal flares. No biologic or JAK-inhibitor therapy had been administered during the reported follow-up period. Her respiratory history was notable for recurrent bronchitis since childhood, daily productive cough, and two episodes of mild hemoptysis. She was heterozygous for a CFTR mutation, without clinical evidence of cystic fibrosis.

High-resolution chest CT showed bilateral bronchiectasis, predominantly involving the lingula, with mucus impaction and tree-in-bud opacities. Diffuse thickening of the tracheal wall and main bronchi was observed, with significant narrowing of the left main bronchus. Flexible bronchoscopy demonstrated diffuse tracheal and bronchial mucosal edema with abundant mucopurulent secretions, cobblestone appearance of the bronchial mucosa, and stenosis of the left main bronchus, still passable with a 5-mm bronchoscope.

Extensive microbiological evaluation of bronchial aspirate and bronchoalveolar lavage was negative. Endobronchial biopsies revealed squamous metaplasia associated with fibrosis and marked chronic inflammatory infiltrates, predominantly lymphoplasmacytic with eosinophilic granulocytes, and evidence of epithelial injury. No granulomas, vasculitides, or malignant cells were identified (Figure 2D). After a multidisciplinary discussion, tracheobronchial involvement as an extraintestinal manifestation of UC was diagnosed.

Systemic corticosteroid therapy with prednisone 50 mg/day was initiated, leading to significant clinical and endoscopic improvement. Pulmonary function tests also showed mild functional improvement following corticosteroid therapy, with FEV1 increasing from 68 to 72% predicted and FVC from 82 to 85% predicted, accompanied by reduction in airway resistance and air trapping. Follow-up bronchoscopy showed reduced mucosal edema, regression of the cobblestone appearance, and increased caliber of the left main bronchus (approximately 9–10 mm), with residual cicatricial stenosis of the lingular bronchus. Temporary discontinuation of steroids due to intolerance raised concerns about recurrence, and the patient remains under close multidisciplinary follow-up.

## Discussion

Tracheobronchial stenosis represents a rare but clinically significant extraintestinal manifestation of ulcerative colitis, posing substantial diagnostic and therapeutic challenges. Although inflammatory bowel diseases are primarily characterized by chronic gastrointestinal inflammation, up to 50% of patients develop systemic manifestations, while pulmonary involvement remains uncommon, traditionally reported in less than 1% of cases (16). However, increasing use of high-resolution imaging and functional respiratory testing suggests that subclinical airway abnormalities may be present in a much larger proportion of patients (17). The three cases presented herein exemplify the heterogeneity of UC-related airway disease and underscore the absence of standardized management strategies, necessitating an individualized, multidisciplinary approach.

Previous case reports have highlighted the broad clinical spectrum of UC-associated pulmonary involvement, which ranges from bronchiectasis to severe inflammatory tracheobronchial disease (18, 19). Alhalabi et al. described a young patient with UC-associated bronchiectasis who presented with chronic cough and mild obstructive ventilatory impairment, and was successfully treated with inhaled corticosteroids and azathioprine (18). This case emphasizes the potential reversibility of early airway inflammation. In contrast, Chakraborty et al. reported a rare case of severe tracheitis and mediastinitis associated with UC, characterized by acute-on-chronic lymphoplasmacytic inflammation and requiring systemic corticosteroids followed by infliximab therapy (19). Consistent with these reports, the present cases demonstrate heterogeneous clinical presentations and treatment responses, ranging from steroid-responsive inflammatory disease to persistent fibrotic bronchial stenosis that required interventional bronchoscopy.

Several shared clinical and pathological features emerge across the cases. All patients were female, ranging from young adulthood to middle age, and in all instances, the left main bronchus was a predominant site of involvement, presenting with significant narrowing or critical stenosis. This anatomical consistency may suggest a predilection for large airway involvement in UC-related respiratory disease, although the underlying pathophysiological mechanisms remain unclear (20). Histopathological findings were also remarkably similar across cases, consistently demonstrating dense chronic lymphoplasmacytic inflammatory infiltrates associated with mucosal edema, epithelial erosions, granulation tissue formation, and variable eosinophilic infiltration (21). Importantly, extensive microbiological investigations were repeatedly negative, supporting an immune-mediated rather than infectious etiology (12).

Another salient feature is the temporal dissociation between intestinal and respiratory disease activity. In two cases, airway involvement developed many years after the initial UC diagnosis, and in one case, it occurred despite previous total colectomy and long-standing gastrointestinal remission. These observations align with prior reports indicating that thoracic manifestations may arise independently of bowel disease activity and, paradoxically, even after “curative” intestinal surgery (9). This further complicates diagnosis, as respiratory symptoms may not be immediately attributed to IBD in the absence of active gastrointestinal disease.

Systemic corticosteroids remain the cornerstone of treatment for UC-related airway involvement, reflecting the shared inflammatory pathogenesis between intestinal and respiratory disease (22). However, the variable response observed in this series highlights the limitations of a uniform medical approach. While one patient experienced rapid and complete clinical improvement, another showed significant but incomplete response with residual cicatricial stenosis, and a third was largely refractory to corticosteroid therapy alone. These differences likely reflect varying degrees of inflammatory versus fibrotic airway remodeling, with advanced structural changes limiting the efficacy of medical therapy (20).

The heterogeneous response to corticosteroid therapy likely reflects differences in active inflammation and established fibrotic remodeling. Some patients improve rapidly after systemic corticosteroids, while others develop persistent cicatricial stenosis despite treatment. These findings suggest that early inflammatory lesions may be reversible, but advanced fibrosis may require interventional bronchoscopic procedures. Rigid bronchoscopy with balloon dilatation can be a valuable option for patients with critical airway obstruction who do not respond to medical therapy (23). Because UC-associated tracheobronchial stenosis is rare, evidence-based management strategies and standardized follow-up protocols are lacking, highlighting the need for individualized multidisciplinary care (24).

Consequently, management strategies diverged considerably among the cases. One patient required rigid bronchoscopy with balloon dilatation due to persistent, critical airway obstruction unresponsive to pharmacological therapy, achieving immediate symptom relief and restoration of airway patency. Another case was managed conservatively with systemic corticosteroids and planned escalation of IBD-directed therapy, emphasizing the importance of controlling systemic inflammation. The third case illustrated an intermediate phenotype, in which inflammation was responsive to steroids but residual fixed stenosis persisted, necessitating long-term surveillance. Together, these experiences highlight that interventional bronchoscopic procedures may be essential in selected patients to prevent irreversible airway compromise.

The absence of evidence-based guidelines remains a major challenge in the management of tracheobronchial involvement in UC (25). Given the rarity of this manifestation, treatment decisions rely on careful exclusion of alternative diagnoses, individualized assessment of disease severity, and close collaboration between pulmonologists, gastroenterologists, and interventional specialists. The characteristic endoscopic appearance—often described as “cobblestone” mucosa—and nonspecific inflammatory histology are suggestive but not pathognomonic, reinforcing that diagnosis is primarily one of clinical correlation (26). This study has several limitations, including the small sample size, retrospective design, lack of control airway samples for histopathological comparison, and the absence of systematic biomarker and longitudinal pulmonary function assessment in all patients. Ultimately, this case series illustrates the broad therapeutic spectrum required to manage UC-related airway disease and underscores the need for heightened clinical awareness and future studies to better define optimal diagnostic and therapeutic pathways.

## Conclusion

In conclusion, these three cases illustrate that, while tracheobronchial stenosis in UC patients often involves similar anatomical targets (the left main bronchus) and similar pathological features (chronic lymphoplasmacytic inflammation), the clinical course and response to treatment are highly variable. The transition from active inflammation to permanent scarring underscores the importance of early recognition to prevent irreversible lung damage. However, until more robust, standardized protocols are developed, the management of this rare EIM will continue to rely on the expertise of multidisciplinary teams tailoring treatments—whether medical or procedural—to the specific needs of the individual patient.

## Data Availability

The original contributions presented in the study are included in the article/supplementary material, further inquiries can be directed to the corresponding author.
